# Chemotherapy dose reduction due to chemotherapy induced peripheral neuropathy in breast cancer patients receiving chemotherapy in the neoadjuvant or adjuvant settings: a single-center experience

**DOI:** 10.1186/2193-1801-3-366

**Published:** 2014-07-16

**Authors:** Bhavana Bhatnagar, Steven Gilmore, Olga Goloubeva, Colleen Pelser, Michelle Medeiros, Saranya Chumsri, Katherine Tkaczuk, Martin Edelman, Ting Bao

**Affiliations:** Department of Internal Medicine, Division of Hematology and Medical Oncology, University of Maryland School of Medicine, 22 S. Greene St N9E24, Baltimore, MD 21201 USA; Department of Pharmacology, University of Maryland School of Medicine, Baltimore, USA; Department of Epidemiology and Biostatistics, University of Maryland School of Medicine, Baltimore, USA

**Keywords:** Peripheral neuropathy, Taxanes, Dose reduction, Breast cancer

## Abstract

**Purpose:**

Taxanes are a cornerstone treatment in early and advanced stage breast cancer and in other common solid tumor malignancies; however, the development of chemotherapy induced peripheral neuropathy (CIPN) often necessitates dose-reduction, which may hamper the effectiveness of the drug and compromise survival outcomes especially when used in the adjuvant setting. Limited literature is available on the prevalence and severity of dose reduction due to CIPN. We sought to determine the frequency and severity of CIPN-induced dose reduction in early stage breast cancer patients who received taxane-based chemotherapy in the neoadjuvant or adjuvant settings.

**Methods:**

We conducted a retrospective single-institution breast cancer clinic chart review of 123 newly diagnosed breast cancer patients and treated with taxane-based neoadjuvant/adjuvant chemotherapy at the University of Maryland Greenebaum Cancer Center between January 2008 and December 2011.

**Results:**

Forty-nine of 123 (40%; 95% CI: 31-49%) patients required dose reduction. Twenty-one (17%; 95% CI: 11-25%) of these patients were dose-reduced specifically due to CIPN that developed during treatment. The median relative dose intensity (received dose/planned dose) for the 21 CIPN-induced dose reduction patients was 73.4% (range, 68.0-94.0%). Patients with diabetes appeared to have a higher risk of taxane-induced dose reduction (*p*-value=0.01). African-American patients and those treated with paclitaxel (rather than docetaxel) experienced a higher-risk of CIPN-induced dose reduction (*p*-values are <0.001 and 0.001, respectively).

**Conclusions:**

The incidence of CIPN-associated dose reduction in our patient population was 17%. African-American patients, diabetics and subjects treated with paclitaxel had a higher risk for CIPN-associated dose reduction in our study.

## Introduction

Chemotherapy induced peripheral neuropathy (CIPN) is a common and potentially dose-limiting complication of many effective cytotoxic agents, including taxanes, which are widely used as part of adjuvant and neoadjuvant doublet and triplet chemotherapy combinations for the treatment of breast cancer. Taxanes function as mictrotubule stabilizing agents (MTSAs) and exert a broad spectrum of cytotoxic effects in cancers of the breast, ovary and lung (Rowinsky & Donehower 1995). The precise pathogenesis through which taxanes exert their neurotoxic effects is unclear; however the underlying mechanism is believed to be promotion of microtubule polymerization and inhibition of depolymerization leading to inhibition of axonal transport within neurons. This results in disruption of signaling in peripheral nerves leading to sensory, motor, or autonomic peripheral neuropathy that may interfere with activities of daily living (ADL) or result in significant disability (Swain & Arezzo 2008). CIPN classically occurs within 24–72 hours following taxane administration and, in most cases is reversible upon prompt discontinuation of the offending agent (Rowinsky et al. 1993; Argyriou et al. 2008). Symptoms are typically described as numbness and tingling in a "stocking-and-glove" distribution, particularly in the distal lower extremities. Patients may also report intermittent sharp, shooting leg pain. Loss of deep tendon reflexes and sensation to pain and temperature are also commonly reported (Argyriou et al. 2008). Previously reported risk factors for CIPN include older age, history of alcoholism, diabetes mellitus, inherited neuropathy, and prior therapy with neurotoxic medications (Makino 2004; Verstappen et al. 2003; Tanabe et al. 2013). In its most severe form, development of CIPN can lead to significant pain syndromes, difficulty with ambulation, and interference with routine daily activities resulting prompt dose reductions and delays that potentially reduce the efficacy of early stage breast cancer treatments (8–10). However, despite the prospect of poorer survival associated with dose reduction, the incidence and severity of dose reduction specifically due to CIPN has not been well-described and studied prospectively. Therefore, we conducted a retrospective chart review evaluating the prevalence, severity, and risk factors associated with dose reduction required due to taxane-induced peripheral sensory neuropathy among breast cancer patients at an academic medical center.

## Materials and methods

This study was approved by the University of Maryland Baltimore Internal Review Board. We reviewed electronic medical and pharmacy records of 123 sequentially treated breast cancer patients who received a taxane as part of neoadjuvant or adjuvant chemotherapy for localized breast cancer between January 1, 2008 and December 31, 2011. Patients were treated per standard of care recommendations and treatment decisions and dose reductions or delays were made based on routine standard of care recommendations. Patients who received treatment on a clinical trial were treated based on the study protocol. Eligible patients were over 18 years of age and had an established diagnosis of non-metastatic breast cancer. Data collected included: demographics (age, race, and gender), taxane received (paclitaxel, nab-paclitaxel, or docetaxel), hormone receptor expression status, HER2/neu expression status, history of diabetes, HIV status, pre-existing peripheral neuropathy, and alcohol history. Progress notes were reviewed to determine whether patients developed peripheral neuropathy during their chemotherapy course and whether dose reductions were instituted at the discretion of the treating physician. Pharmacy records were used to confirm the number of treatment received and the relative dose intensity (received dose/planned dose).

### Statistical analysis

Patients’ demographic and clinical characteristics were assessed and compared between two distinct cohorts of subjects, those who experienced a dose reduction due to any reason and those who did not. In addition we conducted further analyses only among those who experienced a dose reduction, comparing those who had a dose reduction due to CIPN vs. those with dose reductions for other reasons.

We assessed the following potential risk factors for dose-reduction: age, HER2/neu expression status, alcohol abuse, diagnosis of diabetes mellitus, and type of taxane received. Fisher’s and Fisher-Freeman-Halton’s exact tests were used for 2x2 and rxc contingency tables, respectively. All tests were done at the two-sided 0.05 level of significance. Statistical analysis was conducted in SAS version 9.3 (SAS Institute, Cary NC).

## Results

### Patient characteristics

Patient characteristics are shown in Table 1. Of the 123 patients, 120 were women and three were men. The median age at diagnosis was 53 years (range, 32–78). Forty-eight patients (39%) were Caucasian, and 70 (57%) were African-American. Seventy patients received docetaxel, and 46 were treated with paclitaxel. Seven patients either received both agents, or *nab*-paclitaxel. Five patients had a prior history of alcohol abuse, and 20 (16%) had a diagnosis of diabetes mellitus. Forty-two patients (34%) had triple negative breast cancer. Forty nine patients (40%), required dose reduction during the course of their treatment. Twenty one of these (17%) had dose reductions due to CIPN and 28 (23%) had reductions due to other causes. The median relative dose intensity (received dose/planned dose) for the 21 CIPN-induced dose reduction patients was 88% (range, 62%-97%).

**Table 1:** **Selected demographic and baseline characteristics of the patients**

Variable	Patients
**All patients, No. (%)**	123 (100)
**Sex, No. (%)**	
Female	120 (98)
Male	3 (2)
**Race**	
African American	48 (39)
Caucasian	5 (4)
Other	70 (57)
**Age (in years) at diagnosis, median [range]**	53 [32–78]
**Hormone receptor expression status, No. (%)**	
ER+	81 (66)
PR+	67 (54)
HER2/neu overexpressed	16 (13)
**Medical comorbidities, No. (%)**	
DM	20 (16)
HIV	3 (2)
Alcohol abuse	5 (4)
Pre-existing peripheral neuropathy	4 (3)
**Taxane received, No (%)**	
Paclitaxel only	46 (37)
Docetaxel only	70 (57**)**
Multiple agents	7 (6)
**Total patients needing dose reduction, No. (%)**	49 (40)
CIPN	21 (17)
Other causes	28 (24)

### Dose reductions on taxane therapy

Out of 123 total patients, 49 patients (40%) required dose reduction of the taxane they had received. In addition to CIPN, the other common causes for dose reduction included diarrhea (*n* =10), infection (*n*=5), myelosuppression (*n*=5), hypersensitivity reaction (*n*=3), and rash (*n*=2). The distribution of patient characteristics for those who underwent dose reduction and those who did not are shown in Table 2. There were no significant differences between the two groups in terms of tumor characteristics such as estrogen or progesterone receptors expression status or HER2/neu expression, race, age or taxane received. However, patients who experienced toxicities and required a changed regimen mid-treatment, were more likely to have a dose reduction (86% vs. 37%; *p*=0.02). Furthermore, patients with underlying diabetes were more likely to require chemotherapy dose reduction as those without diabetes (65% vs. 35%; *p*=0.02).

**Table 2:** **Selected demographic and baseline characteristics of the patients**

Categorical factor	No dose reduction (***N***=74)	Dose reduction (***N***=49)	***p***-value ^1^
	***N***(row%)	***N***(row%)	
**Her2**			
Negative	64 (63)	38 (37)	0.23
Positive	10 (48)	11 (52)	
**Agent**			
Paclitaxel only	28 (61)	18 (39)	0.57^2^
Docetaxel only	45 (64)	25 (36)	
Multiple agents^3^	1 (14)	6 (86)	0.02^3^
**Alcohol**			
No	71 (60)	47 (40)	1.00
Yes	3 (60)	2 (40)	
**Diabetes**			
No	67 (65)	36 (35)	**0.02**
Yes	7 (35)	13 (65)	
**Race**			
Black	40 (57)	30 (43)	0.57^4^
White	30 (63)	18 (37)	
Other	4 (80)	1 (20)	
**Continuous factor**	**Mean (range)**	**Mean (range)**	
Age (years)	53 (32–78)	55 (35–75)	0.23

^1^ *p-*values calculated with the use of two-sided Fisher’s and Fisher-Freeman-Halton’s tests for categorical variables and the t-test for continuous variables.

^2^Patients treated with paclitaxel (or *nab-*paclitaxel) only vs. those treated with docetaxel only.

^3^Patients treated with multiple agents, compared to those treated with either paclitaxel only or docetaxel only.

^4^Black compared to white only.

### Dose reductions due to CIPN

Patients who were dose-reduced specifically for CIPN were compared to those who were dose-reduced for other causes. These patients were compared according to HER2/neu expression, taxane received, alcohol abuse, history of diabetes, race and age. A greater proportion of African-American (AA) patients had CIPN-related dose reduction (53% vs. 22%; *p*<0.001). Patients treated with paclitaxel appeared to be more likely to require dose reduction due to CIPN than those who received docetaxel (78% vs. 20%; *p*=0.001). No other plausible risk factors were associated with dose reductions due to CIPN. Results of the statistical analyses are summarized in Table 3.

**Table 3:** **Selected demographic and baseline characteristics of the patients grouped by reason for dose reduction**

Categorical factor	Dose reduction due to other reasons (***N***=28)	CIPN dose reduction (***N***=21)	***p***-value ^1^
	***N***(row%)	***N***(row%)	
**Her2**			
Negative	22 (58)	16 (42)	1.00
Positive	6 (55)	5 (45)	
**Agent**			
Paclitaxel only	4 (22)	14 (78)	**0.001** ^**2**^
Docetaxel only	20 (80)	5 (20)	
Multiple agents^3^	4 (67)	2 (33)	0.68
**Alcohol**			
No	27 (57)	20 (42)	1.00
Yes	1 (50)	1 (50)	
**Diabetes**			
No	22 (61)	14 (39)	0.51
Yes	6 (46)	7 (54)	
**Race**			
Black	14 (47)	16 (53)	**<0.001** ^**4**^
White	14 (78)	4 (22)	
Other	0	1 (100)	
**Continuous Factor**	**Mean (range)**	**Mean (range)**	
Age (years)	55 (42–75)	55 (35-67)	0.84

^1^ *p*-values calculated with the use of two-sided Fisher’s and Fisher-Freeman-Halton’s tests for categorical variables and the t-test for continuous variables.

^2^Patients treated with paclitaxel (or *nab-*paclitaxel) only vs. those treated with docetaxel only.

^3^Patients treated with multiple agents, compared to those treated with either paclitaxel only or docetaxel only.

^4^Black compared to white only.

## Discussion

In this single institution retrospective analysis of newly diagnosed breast cancer patients, 17% of patients required dose-reduction due to CIPN, resulting in a median relative dose intensity of 73.4%. These results add to a sparse body of literature pertaining to the frequency of taxane-associated dose reduction. To date, only one other retrospective analysis has described changes in dose due to paclitaxel induced peripheral neuropathy. Among 219 breast cancer patients 4% required discontinuation of treatment as a result of neuropathy (Tanabe et al. 2013), however the number who required dose reduction was not reported.

Dose reduction remains a significant concern as the positive impact of dose intensity of chemotherapy on tumor control of breast cancer has been well-demonstrated (Bonadonna et al. 1995; Budman et al. 1998; Wood et al. 1994). In a study of 207 breast cancer patients who received adjuvant cyclophosphamide, methotrexate and fluorouracil (CMF) chemotherapy, the 20-year overall survival rate was 52% for patients who received ≥ 85% of the total calculated dose for the regimen compared to median overall survival of 11 years for those who received 65-84% of the total calculated dose, and 4.5 years for the patients who received less than 65% of the calculated dose (Bonadonna et al. 1995). Therefore the frequency and risk factors for chemotherapy dose reduction as a result of CIPN is of clinical interest.

In our study, an underlying diagnosis of diabetes mellitus was associated with a two-fold risk for taxane-associated dose reduction. Furthermore, a comparison between patients who were dose-reduced for all causes to those who were reduced for CIPN alone demonstrated that patients treated with paclitaxel were more likely to require dose reduction for CIPN compared to those who received docetaxel and that African-American race appeared to correlate with a higher risk of developing CIPN necessitating dose reduction.

The incidence of peripheral neuropathy due to taxanes varies and is based largely on several predisposing risk factors such as dose per cycle, concurrent therapy with other neurotoxic agents, treatment schedule, cumulative dose, duration of infusion and pre-existing neuropathy from other medical conditions such as diabetes (Argyriou et al. 2008; Lee & Swain 2006; Mielke et al. 2005; Rowinsky et al. 1993). The incidence of paclitaxel-induced severe sensory neuropathy in phase II and III studies is 2-33% for doses 175 mg/m^2^ to 250 mg/m^2^ given every 3 weeks, and 4-24% when given 80 to 100 mg/m^2^ given every week (Gradishar et al. 2005; Jones et al. 2005; Nabholtz et al. 1996; Seidman et al. 1995; Smith et al. 1999; Winer et al. 2004). In most studies, paclitaxel has been reported to result in higher rates of grade 3–4 sensory neuropathy (7-33%) (Winer et al. 2004; Seidman et al. 2008; Smith et al. 1999; Jones et al. 2005) compared to docetaxel (1-9%) (Harvey et al. 2006; Study G et al. 1999; Nabholtz et al. 1999), which may, in part, be related to the vehicle used for paclitaxel administration, Cremophor EL, which has been reported to produce ganglionopathies itself (Mielke et al. 2006). A reported 58% of patients develop, what is referred to as paclitaxel-associated acute pain syndrome (P-APS), a distinct clinical entity recently characterized by Loprinzi and colleagues which may also predispose patients to PIPN later in their treatment course (Loprinzi et al. 2011; Reeves et al. 2012).

Management of CIPN remains challenging considering that commonly used pharmacologic therapies have varying rates of success and often carry their own adverse effects. Gabapentin, nortriptyline, and lamotrigine failed to significantly improve peripheral neuropathy symptoms in patients exposed to taxanes, platinum agents, or vinca alkaloids (Hammack et al. 2002; Rao et al. 2008; North Central Cancer Treatment G et al. 2007). In contrast, duloxetine administration produced modest decreases in the average pain score (1.06 compared to 0.34 for placebo; p-0.003), but was associated with increased fatigue and nausea (Alliance for Clinical Trials in O et al. 2013). Other pharmacologic interventions such as glutamine, vitamin E, amifostine, recombinant human leukemia inhibitory factor have been evaluated for the prevention of CIPN with mixed results (Albers et al. 2011; Postma et al. 1999; Amara 2008; Wang et al. 2007; Stubblefield et al. 2005; Kottschade et al. 2011). Acupuncture has been shown to be a low-risk and well-tolerated non-pharmacological treatment strategy in the management of CIPN, however, its use has been mainly limited to few studies with small numbers of patients (Bao et al. 2011; Zhang et al. 2010; Zhou et al. 2009).

CIPN remains an important toxicity of taxane administration. A recently published study demonstrated that CIPN, in and of itself, has no effect on disease free survival, progression free survival or overall survival (Schneider et al. 2012), however, the potential consequences of dose reduction as a result of CIPN on PFS and OS remain unknown. In its most severe form, CIPN greatly impairs quality of life and can potentially lead to secondary consequences such as increased risk of recurrent falls (Gewandter et al. 2013; Tofthagen et al. 2012). Most importantly, as demonstrated in our report as well as in several others, it can be severe enough to warrant discontinuation of a highly effective class of chemotherapy agents, prompting further investigation for potential risk-factors.

The limitations of this study include the retrospective nature of the data collection, which could result in bias in the determination of cause of dose reduction. In addition, when examining risk factors for CIPN-specific dose reduction, we compared those with CIPN dose reductions to patients who had dose reductions for other reasons. Thus associations may be due to factors that relate to taxane administration, development of CIPN, or other causes. Because CIPN dose reduction is directly related to the severity of CIPN, other comparison groups, such as those who experienced CIPN without dose reduction, would only provide indicators of CIPN incidence or severity. Further elucidation of additional factors for CIPN dose reduction may therefore prove to be problematic.

The strengths of this study include the fact that this is one of the few studies to report incidence and risk factors for dose reduction, as well as the magnitude of dose reduction. In addition, the relatively high sample size and the high number of African Americans in our sample provide further data and increase the generalizability of these results the population of patients undergoing cytotoxic chemotherapy.

In conclusion, CIPN is significant dose-limiting toxicity of taxane use. Elucidation of risk factors will be valuable in identifying patients at risk for developing CIPN and tailoring their treatment accordingly so as to avoid dose reductions of effective chemotherapy agents.
